# Thermal Dynamics of Laser-Irradiated Trilayer Bonded-Zirconia Structures

**DOI:** 10.3390/jfb16040137

**Published:** 2025-04-11

**Authors:** Mitchell Tharp, Jaccare Jauregui-Ulloa, Grace Mendonça De Souza, Susana Salazar Marocho

**Affiliations:** 1 Biomedical Materials Science Department, School of Dentistry, University of Mississippi Medical Center, Jackson, MS 39216, USA; mtharp3@umc.edu (M.T.); jjaureguiulloa@umc.edu (J.J.-U.); 2 Restorative Dentistry Department, School of Dental Medicine, University at Buffalo, Buffalo, NY 14214, USA; gdesouza@buffalo.edu

**Keywords:** zirconia, heat transfer, temperature mapping, bonding

## Abstract

This study aims to assess the thermal dynamics of supporting structures during laser-assisted debonding of bonded yttrium-stabilized zirconia (YSZ) ceramic. We tested the hypothesis that the heat transfer to dentin analog material and composite resin resembles that of dentin. Thirty sintered YSZ (ZirCAD, Ivoclar, Schann, Liechtenstein) slabs (4 mm diameter, 1 mm thickness) were air particle abraded, followed by two coats of Monobond Plus (Ivoclar). The slabs were bonded to exposed occlusal dentin, NEMA G10 dentin analog, or composite resin cylinders using Multilink Automix (Ivoclar) dual-cured cement. The bonded YSZ specimens (n = 10/group) subjected to irradiation with an Er,Cr:YSGG laser (Waterlase MD, Biolase, Foothill Ranch, CA, USA) at 7.5 W, 25 Hz, with 50% water and air for 15 s. Heat transfer during laser irradiation was monitored with an infrared camera (Optris PI 640, Optris GmbH, Berlin, Germany) at 0.1-s intervals. Data were analyzed using one-way ANOVA, which showed no significant differences in mean temperature between zirconia and cement layers across the substrates (composite resin, G10, dentin) (*p* = 0.0794). These results suggest flexibility in substrate choice for future thermal dynamics studies under laser irradiation.

## 1. Introduction

An escalating demand for strong and aesthetically pleasing restorations among patients has led to the development of new all-ceramic materials, most notably yttrium-stabilized zirconia (YSZ) restorations. YSZ ceramics can be milled into monolithic restorations or into frameworks. Different types of YSZ ceramics are used in dental restorations, such as 3 mol% yttria-stabilized tetragonal zirconia polycrystal ceramic (3Y-TZP) which is a specific and popular variant in the field. The 3Y-TZP ceramic is favored for its superior fracture toughness compared to other YSZ ceramics. This property is attributed to their transformation toughening mechanism. Such mechanisms consist of the transformation of small tetragonal to large monoclinic (t → m) grains due to a stress-induced mechanism, resulting in crack arresting and increased toughness of the material [1]. YSZ crowns provide superior longevity and lower rates of fracture when compared to their porcelain-fused-to-metal counterparts [2]. These characteristics have positioned YSZ as a material of choice for both anterior and posterior restorations. However, inherent complications in YSZ restorations arise from the materials’ exceptional mechanical properties, particularly the challenge of removing the restoration when necessary. Indeed, in general, any all-ceramic restorations can fail due to biological, technical, or mechanical reasons. Biological factors commonly include the presence of underlying decay, the need for root canal treatment or retreatment, and biological width invasion, among other factors. Technical failures can result from irreparable ceramic chips, fractures, color or shade mismatches, and poor marginal adaptation. Mechanical failures are often stress-induced, including fractures from prolonged occlusal overload, or structural weakness due to inadequate design. In such situations, the removal of the all-ceramic restoration becomes necessary. Restoration removal techniques vary in their approach and impact on the restoration. Furthermore, adhesively bonded ceramic restorations carry the added risk of fracturing the underlying tooth structure during removal. The ideal method for ceramic removal should be non-traumatic and efficient, and should preserve the integrity of the supporting structure. While the literature discusses various devices and techniques, many of these methods have not been updated in around 20 years, and not all of them are suitable for all-ceramic restorations [3,4,5]. Some methods aim to preserve the restoration intact for potential reuse, typically by applying traction or controlled percussion to break the bond with the non-adhesive luting cement. These include ultrasonic energy, pneumatic instruments, sliding hammers, and crown tractors. Other techniques allow for restoration removal with minor damage, making repairs possible. These methods involve creating a small access point, such as a slot or hole, in the restoration to facilitate controlled force application between the restoration and the underlying tooth structure. In cases where removal without significant damage is not feasible, sectioning the restoration and removing it in pieces is required. This is, in fact, the most conventional method for removing all-ceramic restorations. The other methods mentioned are unsuitable for adhesively bonded all-ceramic restorations, as the strong bond increases the risk of fracturing the underlying tooth structure during removal, which can also damage the periodontium due to the forces involved, making non-destructive techniques less effective [4]. Conventional means, such as using a diamond or tungsten bur to section and remove an all-ceramic crown, have proven to be both arduous and time-consuming, especially when performed on YSZ restorations [5,6,7]. This has sparked increased interest in non-mechanical instrumentation. The recent literature indicates that laser energy, when transmitted in the form of heat through the YSZ crown, can reach the ceramic–cement interface, weaking the bond strength between the YSZ and the underlying cement by causing softening. Indeed, the laser method is a viable and effective approach for all-ceramic type crown removal [8]. Various lasers and settings have been explored for this purpose, with erbium-based lasers, such as Erbium-doped Yttrium Aluminum Garnet (Er:YAG) and Erbium, Chromium-doped Yttrium Scandium Gallium Garnet (Er,Cr:YSGG) showing effective results. These erbium lasers have been tested with different settings for the removal of a range of ceramic restorations, from glass-ceramics to polycrystalline ceramics [9,10,11,12,13,14,15,16,17,18,19,20]. Clinical case reports have demonstrated the effectiveness of erbium lasers in removing adhesively bonded YSZ and partially stabilized zirconia (PSZ) restorations [10]. For these high-strength polycrystalline zirconia-based ceramics, Er:YAG lasers have been evaluated with energy settings ranging from 300 to 590 mJ, power levels of 4.5 W, and frequencies between 10 and 15 Hz [9,10,18,19,20]. Similarly, the energy settings for Er,Cr:YSGG lasers have been assessed at power levels between 4.5 and 6 W with frequencies ranging from 15 to 20 Hz [9,10,18]. Higher power settings have resulted in shorter debonding times, whereas lower power settings have prolonged the process [20]. Most studies have focused on evaluating the debonding time of YSZ crowns under laser irradiation [18,19,20]. An important aspect of YSZ restorations is their low thermal conductivity [21], which means it does not effectively transfer heat. As a result, the thermal effects from laser usage are expected to remain localized rather than spread throughout the material. However, despite YSZ’s poor heat conduction, prolonged or high-intensity erbium-based laser exposure can still generate sufficient heat at the ceramic–cement interface to favor debonding, as mentioned previously. While some heat transfer to the underlying substrate is necessary for debonding, excessive heat beyond the cement microlayer could pose a risk to the pulp if excessive temperatures thresholds are exceeded. While it is well established that temperatures exceeding 44 °C can be detrimental to pulpal tissue, causing irreversible damage such as inflammation or necrosis [22], the temperature dynamics across the different substrates of a bonded restoration, such as dentin, pulp, and even analog dentin, have not been being sufficiently explored. Therefore, understanding how heat is transferred through the substrates during laser-assisted debonding is essential. Investigating the thermal responses induced by high-powered lasers is important not only for ensuring effective restoration removal but to prevent unintended thermal damage to the underlying structures.

Dentin and composite resin, or a combination of both, are two of the most frequent indirect restoration substrates. These substrates exhibit distinct physical properties and composition. Dentin is composed of approximately 70% inorganic material, primarily hydroxyapatite crystals. Dentin has a higher organic content compared to enamel, making up about 20% of its composition. This organic material includes collagen fibers, proteins, and 10% water. Dentin serves as a protective and support structure capable of absorbing chewing stresses. The dentinal tubules transmit sensory stimuli, such as temperature and pressure to the pulp, contributing to tooth sensitivity. Dentin’s inherent moisture content can pose challenges for adhesive bonding procedures, which are especially relevant when considering restoration durability and debonding dynamics. Dentin exhibits approximately 4.2 times lower hardness (274.8 ± 18.1 HV vs. 65.6 ± 3.9 GPa) [23] and a lower elastic modulus (1.3–4.9 GPa vs. 39.5–108.2 GPa) [24] compared to enamel. By contrast, composite resin-based materials (RBCs) are synthetic materials composed of a resin matrix and inorganic fillers, with no water content. Adjusting the ratio of these components enables tailoring of physical properties such as hardness and wear resistance. For instance, microhybrid composite resins are considered universal composites due to their satisfactory clinical performance in both anterior and posterior teeth. Their fillers typically average about 0.4–1.0 μm [25] and exhibit high hardness [26] and elastic modulus [27]. These RBCs can be used as a core build-up material to restore the coronal portion of the teeth and to achieve retention and resistance form for the crown.

NEMA G10, a woven glass fiber-filled epoxy resin, is used as a dentin analog material in dental materials research due to its similar elastic properties and bond strength to resin-based cement when compared to natural hydrated dentin. Both G10 and dentin exhibit a highly linear elastic modulus and indistinguishable stress–strain behavior. These similarities highlight G10’s suitability for applications that require close approximation to the natural characteristics of dentin [28]. Additionally, the consistent bond strength of G10 under both dry and wet conditions further reinforces G10’s suitability as a dentin substitute in these studies, allowing for accurate simulation of clinical scenarios [28]. Therefore, findings obtained using G10 can reliably be extrapolated to a dentinal substrate, making G10 particularly relevant for investigating in vitro bonding and debonding processes.

This study examined thermal changes through a YSZ ceramic adhesively bonded to a resin-based cement and one of the following three substrates: dentin, dentin analog material (NEMA G10), and composite resin. The G10 was chosen as a substrate in this study as it is commonly used for research purposes as a substitute for dentin due to its similar organic composition and mechanical properties [28,29]. By comparing these three substrates, this study provides insights into how thermal energy from laser applications interacts with different materials and through the bonding interfaces. Understanding the thermal behavior across dentin, its analog G10, and composite resin can help tailor laser parameters to minimize pulpal risk while maintaining effective debonding. Furthermore, identifying whether G10 and composite resin accurately mimic dentin under laser conditions supports the broader applicability and validity of in vitro studies using these materials. We specifically aimed to test this null hypothesis, that the thermal response of the dentin analog material and composite resin resembles that of dentin.

## 2. Materials and Methods

A YSZ block (e.max ZirCAD, MO 0, Ivoclar) was symmetrically sectioned into small cuboids, shaped into cylinders with a 6.6 mm diameter, and then sliced to produce nine rounded slabs. After sintering, following the manufacturer’s recommendations (Zircomat, Ivoclar), the slabs had final dimensions of 4 mm in diameter and 1 mm in thickness, and were subjected to air particle abrasion with 30 µm silica-coated aluminum oxide particles (Rocatec, 3M) and subsequently cleaned in an ultrasonic bath for five minutes. After air drying the YSZ slabs, two coats of a silane-based primer (Monobond Plus, Ivoclar) were applied, and the slabs were bonded onto either the exposed occlusal dentin of a maxillary premolar, a NEMA G10 cylinder, or a composite resin cylinder using a dual-cured cement (Multilink Automix, Ivoclar).

**Figure 1:**
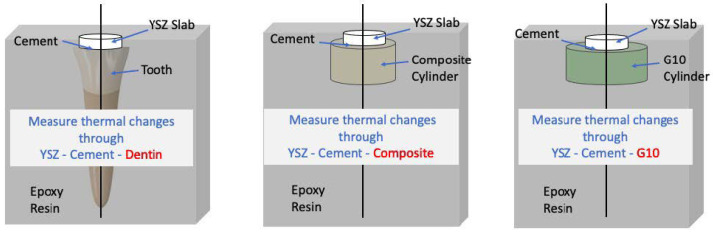
Schematic of the bonded samples and tested groups. The black line delineates where the bonded samples were cross-sectioned to reveal the entire diameter of the bonded interface. This enabled tracking of temperature changes using the IR camera.

To obtain the flat dentin surface, the coronal surface of the premolar teeth was first sliced and ground to expose the dentin, followed by polishing with 600-grit silicon carbide paper. Prior to and after slicing, the teeth were examined with an optical microscope (Keyence, Osaka, Japan) to ensure no enamel remained in the area to be bonded. The dentin was then treated with acid etching, using 37% phosphoric acid (Best Etch, Vista Apex, Racine, WI, USA), rinsed, and dried as preparation for the subsequent cementation procedure. The resulting sets were encased in epoxy resin and subsequently sagittally sectioned using an automated cutting machine (Accutom-50; Struers, Ballerup, Denmark), producing 30 samples total (ten per substrate) to undergo laser irradiation (Figure 1).

A G10 rod stock with a diameter of 12.8 mm was cut to a height of 5 mm to serve as the bonding substrate. To prevent any dehydration and ensure the material stayed stable for the next steps, the 5-mm thick slices were soaked in distilled water for two weeks. The G10 surfaces were ground using 600-grit silicon carbide paper under irrigation, followed by acid etching with 5% hydrofluoric acid (IPS Ceramic etching gel, Ivoclar) for 20 s. The surfaces were rinsed, dried, and treated with a silane-based primer (Monobond Plus, Ivoclar) in preparation for the bonding process.

**Figure 2:**
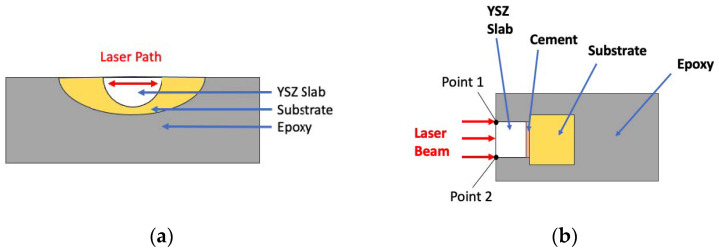
(**a**) Top view of a representative cross-section of a bonded YSZ–cement–substrate sample showing the laser irradiation path. (**b**) IR camera schematic view of the trilayer sample. The laser beam was irradiated from point 1 to 2.

The samples were irradiated using an erbium,chromium:yttrium–scandium–gallium–garnet (Er,Cr:YSGG) laser (Waterlase MD, Biolase) at 7.5 W, 25 Hz, and 50% water and 50% air for 15 s, consisting of five strokes, each lasting three seconds, in a straight back-and-forth motion, tracing the width of the zirconia–cement bonded interface along the plane of the sagittal cut (Figure 2) with a sapphire tip (MG6; Biolase, Foothill Ranch, CA, USA).

The maximum temperatures in the bonded structures (YSZ–cement–substrate) during laser irradiation were monitored and recorded every 0.1 s using an infrared camera (IR, Optris PI640, Optris GmbH, Berlin, Germany). The camera was positioned at a fixed distance of 50 mm from the sample to ensure consistent and accurate thermal measurements. During data acquisition, a square region of interest (ROI) was drawn on the thermal images to focus on specific areas of the sample, such as the YSZ slab, cement, and substrate, as shown in Figure 2. The camera’s high-resolution detector (640 × 480 pixels) enabled detailed tracking of temperature variations. Real-time thermographic images were recorded continuously to monitor temperature changes through the trilayer sets. With its adaptive frame rate, the IR camera dynamically adjusts the number of measurements captured per unit time based on emissivity, and temperature variations. When the scene changes rapidly, the camera increases its sampling rate to capture finer temperature fluctuations with greater precision. The data was analyzed using one-way ANOVA. Temperature variation profiles for each trilayer combination are presented in the graphs.

## 3. Results

**Figure 3:**
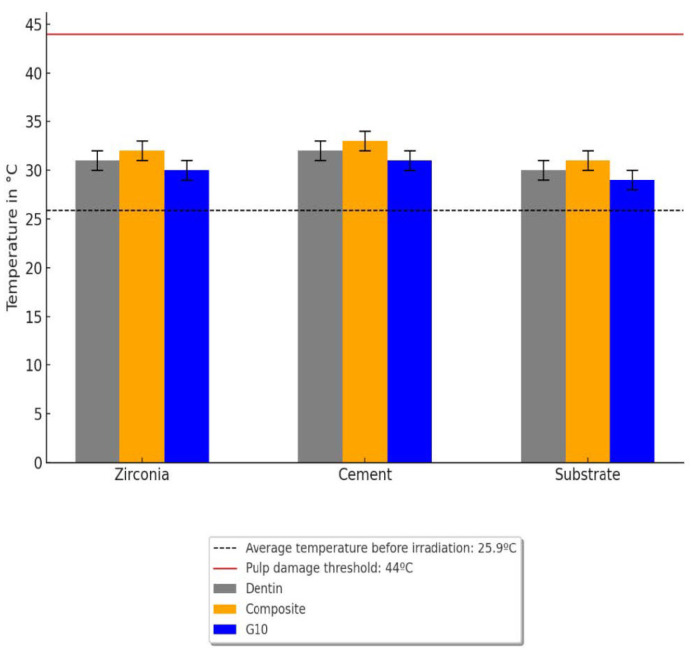
Mean thermal values obtained during laser irradiation for each layer.

**Figure 4:**
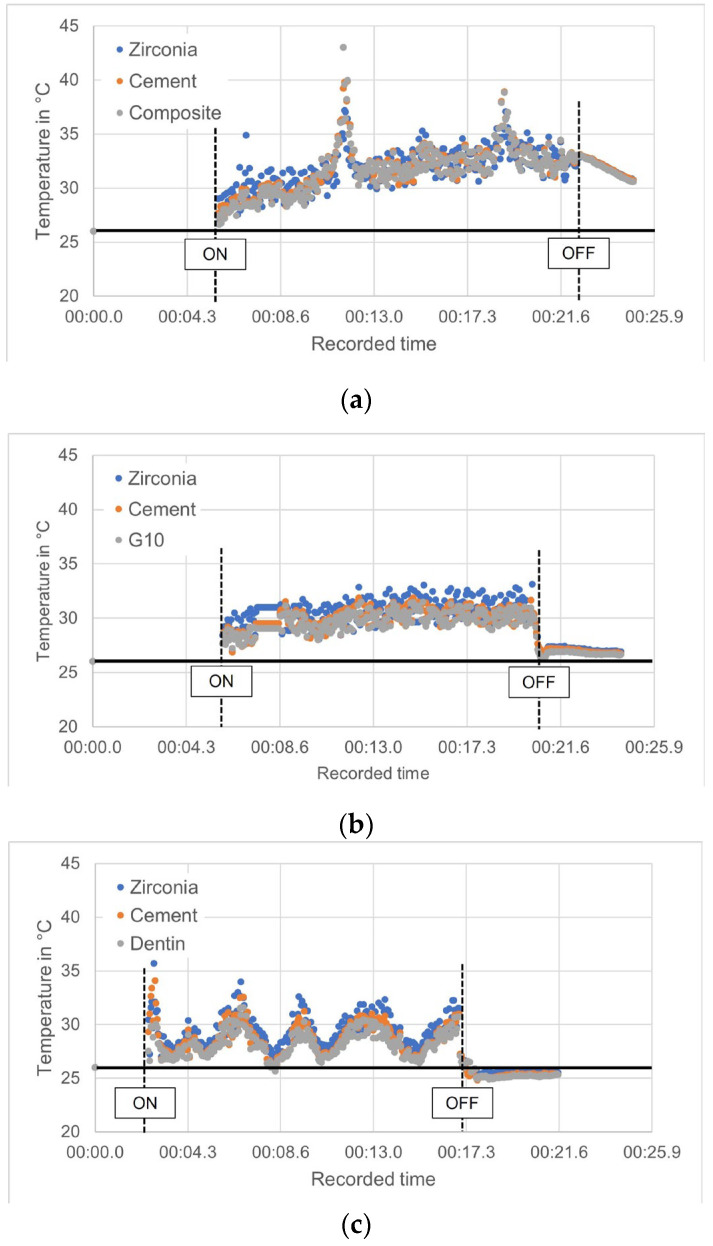
Temperature changes induced by the laser in the ROI of the trilayer set when the substrate was (**a**) composite resin, (**b**) G10, and (**c**) dentin. The black horizontal line represents the base temperature. The vertical dashed lines mark the moment when the laser was activated (ON) and subsequently turned off (OFF) after 15 s.

The power analysis indicated a 100% power level with ten samples tested per group. One-way ANOVA of the temperature data indicated no statistically significant difference in the average temperature across the different layers (zirconia and cement) with any substrate (composite resin, G10, and dentin) (*p* = 0.0794). There was also no significant difference between the substrates, with all average thermal values between 30.6 °C and 32.7 °C. The results are summarized in Figure 3. The temperature profile, from the moment the laser beam is turned on for irradiating the bonding interface until it is turned off, is shown in Figure 4a–c. Before the laser was activated, the base temperature remained stable at ~26 °C. Upon laser irradiation, a noticeable temperature increase occurred during the 15-s laser exposure. For the sets bonded to G10 and dentin, the heat dissipated rapidly once the laser was turned off, with no sustained elevation in temperature observed beyond the irradiation period. The temperature rise was transient, and no significant thermal buildup was recorded post-irradiation for these materials. The rise in temperature had fluctuations. Analyzing the data points, a brief, non-sustained temperature spike occurs within 0.1 s. However, the overall mean maximum temperature remained below 35 °C throughout laser irradiation, remaining well below critical thresholds that could compromise pulpal health. After the laser was turned off, the temperature began to drop rapidly in the sets with G10 and dentin substrates, though cooling was delayed in the trilayer sets with a dental composite substrate. The temperature returned to baseline within a short period, although in some cases, a slightly elevated temperature may persist for a brief time.

## 4. Discussion

Erbium-based lasers are a promising alternative technique to remove all-ceramic restorations, such as monolithic feldspathic, lithium disilicate, and zirconia crowns, compared to diamond burs in a high-speed handpiece. The debonding with these lasers generally does not impart damage to the ceramic restoration, allowing for a less invasive and less traumatic patient experience, and the potential reuse of the restoration if needed [5,10,12], which can significantly reduce both clinical chair time and overall financial costs for the patient and clinician. A retrospective case series evaluating the effectiveness of the Er,Cr:YSGG lasers, reported a high success rate of over 95% for intact debonding of ceramic prostheses. The adhesively bonded restorations examined consisted of 13 lithium disilicate and 39 zirconia units, including veneers, single crowns, and three fixed dental prostheses (FDP). Lithium disilicate veneers (2.25 ± 0.61 min) and crowns (5.9 ± 2.4 min) were generally faster to remove than monolithic CAD/CAM zirconia crowns (7.1 ± 8.9 min) [10]. This difference in removal time likely can be attributed to the fact that lithium disilicate allows for greater laser transmission compared to the polycrystalline zirconia ceramic. As a result, the energy absorption at the cement interface is more efficient, reducing the time needed for debonding. These distinctions emphasize the importance of material-specific considerations when selecting laser settings and treatment planning protocols.

When erbium-based lasers are used with high-strength dental ceramics, like zirconia-based ceramics, these ceramics primarily transmit energy rather than absorb it. This phenomenon forms the foundation for laser debonding techniques, wherein the cement beneath a restoration can absorb the transmitted energy, resulting in decreased bonding strength [30]. Rechmann et al. [31] demonstrated that the energy absorption band of multiple resin-based cements corresponds to erbium laser energy emissions, as the cements showed an H_2_O/OH absorption band, which coincides with the erbium-based laser wavelength. Research such as this highlights the mechanism of action of the erbium-based laser in debonding dental ceramics from the resin-based cement. The laser’s energy is absorbed by the organic components within the resin. This absorption triggers an ablation process characterized by explosive vaporization, followed by hydrodynamic ejection. The rapid heating and melting of the organic components generate significant expansion forces due to the material’s volume change upon melting [32]. This process, known as thermal ablation, effectively breaks the bonds within the cement, facilitating the removal of the ceramic restoration. Additionally, the continuous water spray used during laser irradiation helps to control the temperature rise and prevents overheating, further enhancing the safety of the procedure, and explaining why dry lasering is not advisable [5]. The novel information in our study relates as well to the Er,Cr:YSGG laser temperature sustainability. Tracking temperature changes during laser activation and deactivation revealed that cooling was delayed in the trilayer sets with composite resin as a substrate, indicating a potential heat retention effect. Unlike dentin and G10, composite resin exhibits a lower thermal conductivity, which may slow down heat dissipation after laser irradiation.

While the maximum temperature remained below critical thresholds across all tested sets, the transient increase and delayed cooling in composite-based sets could be relevant in clinical settings where cumulative effect of repeated or prolonged laser exposure occurs. The absorption and dissipation of laser energy are influenced by multiple factors, including resin composition, degree of polymerization, and filler content. Some composite resins may retain heat longer due to variations in their filler-to-matrix ratio, affecting overall thermal behavior. In practical applications, this may necessitate adjustments in laser settings, such as pulse duration or cooling intervals, to optimize safety and efficiency. Prolonged or repeated exposure may lead to cumulative thermal effects, necessitating careful monitoring. Deeb et al. [19] assessed the effectiveness of Er,Cr:YSGG in debonding prefabricated zirconia crowns (NuSmile), reporting an average debonding time of approximately 2 min and 34.7 s. While the study confirmed the efficiency of laser-assisted debonding, the duration of the process remains a noteworthy concern. Extended laser exposure could contribute to localized heating, especially within composite substrates, potentially affecting the underlying tooth structure and pulp. Future studies should explore the impact of prolonged laser energy absorption within the composite material to better understand these thermal dynamics and refine laser-assisted debonding protocols for different substrate materials. Additionally, Deeb et al. reported a mean temperature increase of 2.2 ± 0.9 °C induced by the Er,Cr:YSGG laser when used with a continuous water spray, confirming that it operates within a safe temperature range, and aligns with the results of the present study regarding the thermal impact on the substrate level below the bonding interface. This finding highlights the importance of controlling the rise in temperature by adhering to proper cooling protocols, such as water spray, during laser applications to prevent potential thermal injury to the dental pulp.

Further research is essential to optimize the laser parameters, such as power, pulse duration, and frequency, to enhance the efficiency of the debonding process while minimizing the risk of thermal damage. The variability in debonding time observed by Deeb et al. [10,11,19] suggests that individual factors, such as the laser parameters, the thickness and type of the ceramic material, the shade and opacity of the ceramic, and the type and thickness of the resin cement, can influence outcomes. Therefore, tailored laser settings for different clinical scenarios could improve the predictability and success of laser-assisted ceramic debonding. The detailed clinical studies on the use of the Er,Cr:YSGG laser for debonding dental ceramics have demonstrated its potential as a viable alternative to traditional rotary instruments. The laser’s ability to efficiently and safely remove ceramic restorations without causing significant thermal or structural damage to the tooth or restoration makes it an attractive option for both clinicians and patients, as its unique potential to preserve the integrity of the restoration allows for possible reuse, resulting in reduced chair time and meaningful cost savings. As research continues, the development of standardized protocols and optimized laser settings will further enhance the clinical application of the Er,Cr:YSGG laser in dental practices. Although the erbium-based lasers have proved their effectiveness for debonding zirconia restorations, the bonded substrates are rarely investigated in terms of thermal or structural changes. This study revealed that the energy transmitted through zirconia, resin-based cement, and substrate remained below the thermal threshold that causes pulpal damage. Additionally, it demonstrated that temperature changes were consistent across all trials, irrespective of the substrate involved, whether it be dentin, G10, or composite resin.

Despite the differences in physical properties between composite resin and dentin, they possess comparable thermal conductivity properties. While the coefficient of thermal expansion (CTE) of composite resin (26 to 35 × 10^−6^/°C) [32] and dentin (11 × 10^−6^/°C) [33] are different, their thermal response to laser irradiation is influenced by several factors beyond just their CTE. Both composite resin and dentin demonstrate similar absorption coefficients for laser wavelengths ranging from 405 to 650 nm [34]. This means they absorb similar amounts of laser energy, leading to comparable thermal responses. A resemblance in this parameter supports our findings that dentin and composite resin behaved similarly during laser irradiation without excessive heat accumulation that could be detrimental to pulp vitality. As previously mentioned, G10 shares a similar composition profile with dentin. It has frequently been used in dental material studies since Kelly et al. [28] found that G10 was not significantly different from hydrated dentin, in terms of blunt contact elastic behavior or resin cement bond strength. Given this context, the thermal behavior of G10 in this study aligns closely with that of dentin. Because of this, G10 could be used as an alternative material to dentin to assess thermal behavior, where limited extracted teeth are available for laboratory testing. The temperature fluctuations observed during laser irradiation (Figure 4) over time could be attributed to thermal feedback mechanisms in which, as the material heats up, there might be feedback loops where the temperature changes influence the way the material absorbs or reflects the laser energy, resulting in temperature fluctuations. However, further studies should be conducted to determine whether the thermal conductivity and diffusion properties of G10 and dentin are comparable under different laser parameters and wavelengths. Further research could focus on clinically relevant specimen configurations to enhance the applicability of these findings.

A key area for further exploration in this study is the simulation of fluid dynamics within the pulp of a live tooth, which represents a limitation in our current work. Another approach to better understand how laser irradiation impacts the underlying tooth structure, beyond dentin, would be incorporating simulations of the pulp tissue and using longer irradiation times to simulate a clinical situation. Furthermore, irradiating crowns within this model would enhance the relevance of the findings. Addressing these aspects could enhance the understanding of various factors in the laser debonding process, such as heat dissipation, and thermal conductivity. Future research that incorporates these dynamics could provide a more comprehensive view and lead to improved techniques in laser debonding.

## 5. Conclusions

The results indicate that the substrates studied exhibited comparable thermal behavior under laser irradiation, with one notable exception: the composite resin substrates showed a delayed cooling response. This finding is significant as it suggests flexibility in choosing substrates for future in vitro studies assessing thermal behavior under laser irradiation conditions. Specifically, the data indicates that both composite resin materials and dentin analog substrates, like G10, exhibit thermal behavior akin to dentin, although composite resin may experience a slower cooling rate when subjected to laser irradiation. It is important to take substrate thermal properties into account when selecting materials for laser-based procedures. Developing refined protocols that address these differences will help ensure both safety and effectiveness in clinical applications.

An important area for additional exploration includes simulating the fluid dynamics within the pulp of a live tooth. Future work should include pulp tissue simulations and longer irradiation times to better mimic clinical conditions and assess the impact on underlying tooth structures beyond dentin.

## Data Availability

The data presented in this study are available on request from the corresponding author. Due to institutional limitations, public access is not available at this time.

## Abbreviations

The following abbreviations are used in this manuscript:

YSZ	Yttria Stabilized Zirconia
Er,Cr:YSGG	Erbium,Chormium:yttrium–scandium–gallium–garnet
FDP	Fixed dental prostheses
CAD/CAM	Computer aided design/Computer aided manufacturing
